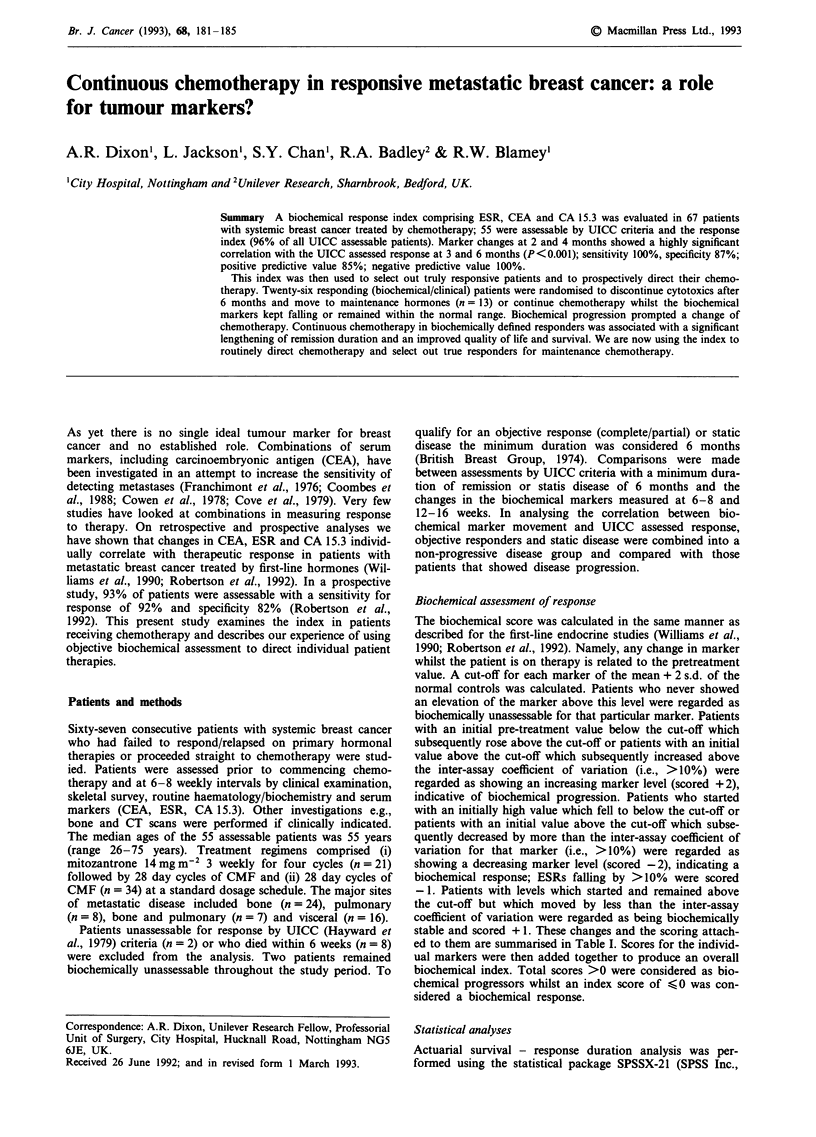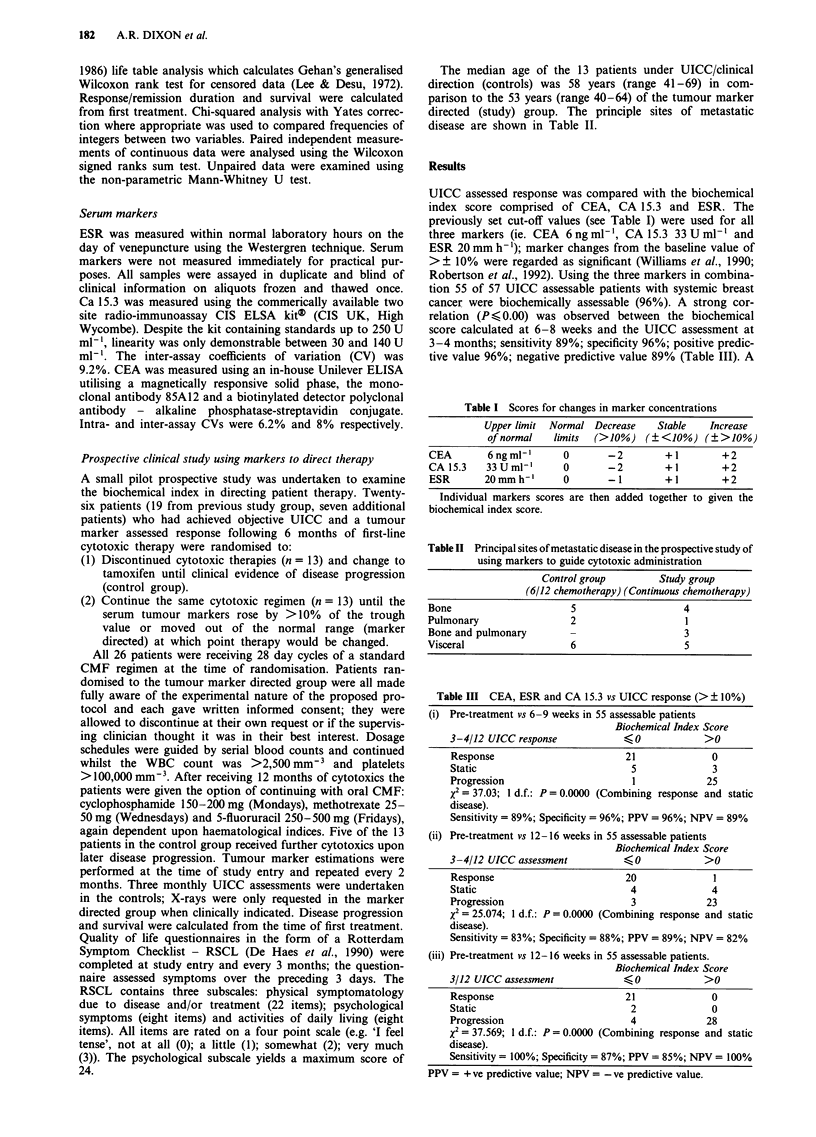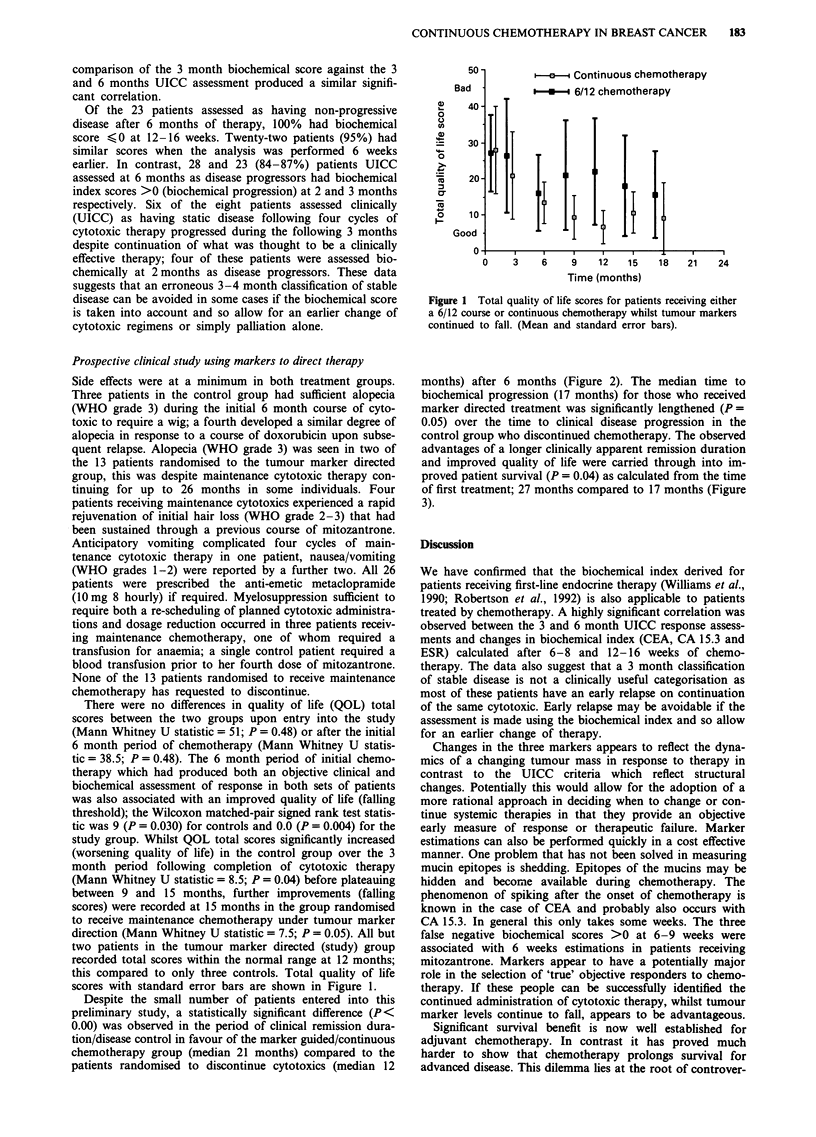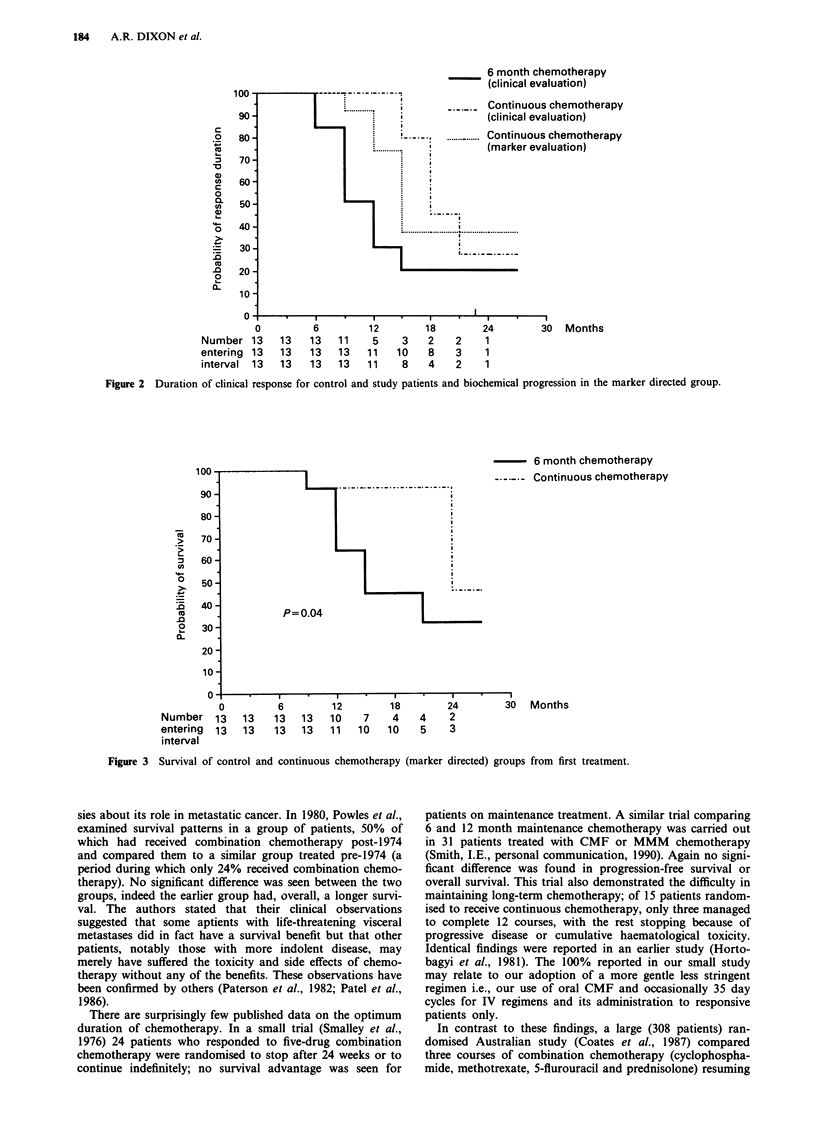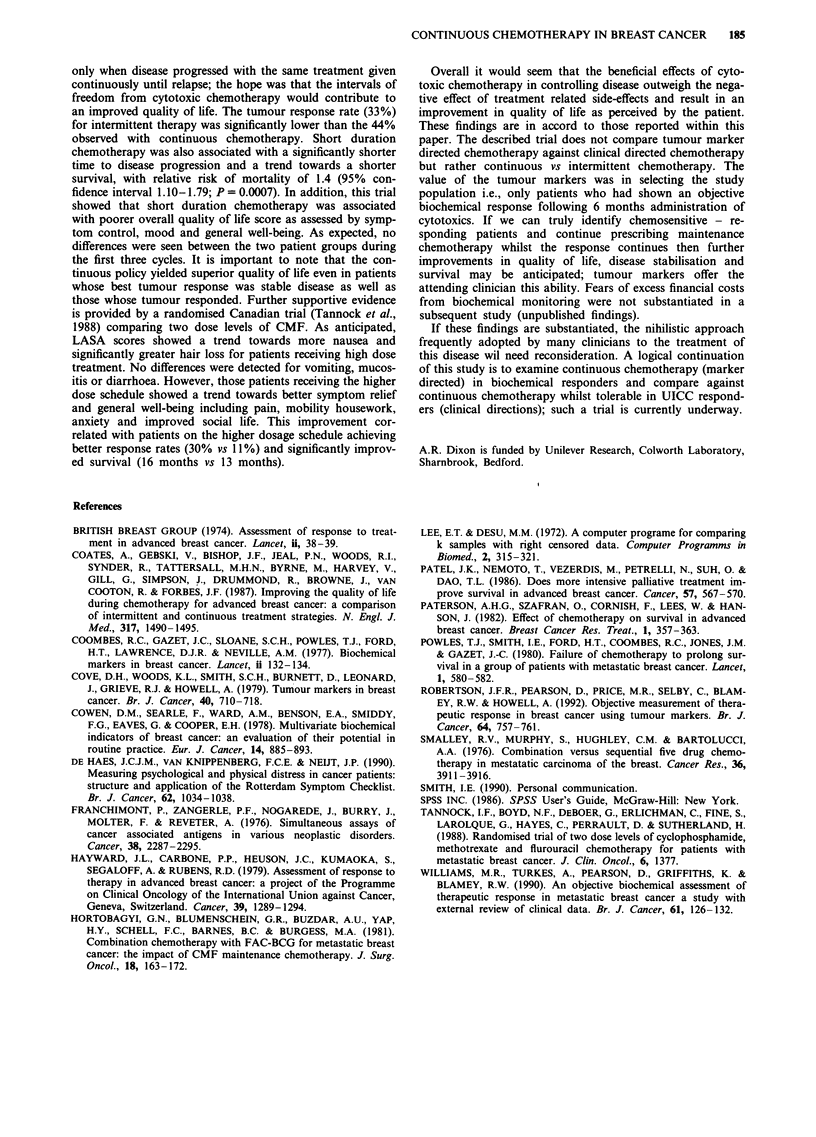# Continuous chemotherapy in responsive metastatic breast cancer: a role for tumour markers?

**DOI:** 10.1038/bjc.1993.310

**Published:** 1993-07

**Authors:** A. R. Dixon, L. Jackson, S. Y. Chan, R. A. Badley, R. W. Blamey

**Affiliations:** City Hospital, Nottingham, UK.

## Abstract

A biochemical response index comprising ESR, CEA and CA 15.3 was evaluated in 67 patients with systemic breast cancer treated by chemotherapy; 55 were assessable by UICC criteria and the response index (96% of all UICC assessable patients). Marker changes at 2 and 4 months showed a highly significant correlation with the UICC assessed response at 3 and 6 months (P < 0.001); sensitivity 100%, specificity 87%; positive predictive value 85%; negative predictive value 100%. This index was then used to select out truly responsive patients and to prospectively direct their chemotherapy. Twenty-six responding (biochemical/clinical) patients were randomised to discontinue cytotoxics after 6 months and move to maintenance hormones (n = 13) or continue chemotherapy whilst the biochemical markers kept falling or remained within the normal range. Biochemical progression prompted a change of chemotherapy. Continuous chemotherapy in biochemically defined responders was associated with a significant lengthening of remission duration and an improved quality of life and survival. We are now using the index to routinely direct chemotherapy and select out true responders for maintenance chemotherapy.


Br. J. Cancer (1993), 68, 181–185 © Macmillan Press Ltd., 1993

### Continuous chemotherapy in responsive metastatic breast cancer: a role for tumour markers?

A.R. Dixon[1], L. Jackson[1], S.Y. Chan[1], R.A. Badley[2] & R.W. Blamey[1]

[1]*City Hospital, Nottingham and* [2]*Unilever Research, Sharnbrook, Bedford, UK.*

**Summary** A biochemical response index comprising ESR, CEA and CA 15.3 was evaluated in 67 patients with systemic breast cancer treated by chemotherapy; 55 were assessable by UICC criteria and the response index (96% of all UICC assessable patients). Marker changes at 2 and 4 months showed a highly significant correlation with the UICC assessed response at 3 and 6 months ($P<0.001$); sensitivity 100%, specificity 87%; positive predictive value 85%; negative predictive value 100%.

This index was then used to select out truly responsive patients and to prospectively direct their chemotherapy. Twenty-six responding (biochemical/clinical) patients were randomised to discontinue cytotoxics after 6 months and move to maintenance hormones ($n = 13$) or continue chemotherapy whilst the biochemical markers kept falling or remained within the normal range. Biochemical progression prompted a change of chemotherapy. Continuous chemotherapy in biochemically defined responders was associated with a significant lengthening of remission duration and an improved quality of life and survival. We are now using the index to routinely direct chemotherapy and select out true responders for maintenance chemotherapy.

As yet there is no single ideal tumour marker for breast cancer and no established role. Combinations of serum markers, including carcinoembryonic antigen (CEA), have been investigated in an attempt to increase the sensitivity of detecting metastases (Franchimont *et al.*, 1976; Coombes *et al.*, 1988; Cowen *et al.*, 1978; Cove *et al.*, 1979). Very few studies have looked at combinations in measuring response to therapy. On retrospective and prospective analyses we have shown that changes in CEA, ESR and CA 15.3 individually correlate with therapeutic response in patients with metastatic breast cancer treated by first-line hormones (Williams *et al.*, 1990; Robertson *et al.*, 1992). In a prospective study, 93% of patients were assessable with a sensitivity for response of 92% and specificity 82% (Robertson *et al.*, 1992). This present study examines the index in patients receiving chemotherapy and describes our experience of using objective biochemical assessment to direct individual patient therapies.

### Patients and methods

Sixty-seven consecutive patients with systemic breast cancer who had failed to respond/relapsed on primary hormonal therapies or proceeded straight to chemotherapy were studied. Patients were assessed prior to commencing chemotherapy and at 6–8 weekly intervals by clinical examination, skeletal survey, routine haematology/biochemistry and serum markers (CEA, ESR, CA 15.3). Other investigations e.g., bone and CT scans were performed if clinically indicated. The median ages of the 55 assessable patients was 55 years (range 26–75 years). Treatment regimens comprised (i) mitozantrone 14 mg m$^{-2}$ 3 weekly for four cycles ($n = 21$) followed by 28 day cycles of CMF and (ii) 28 day cycles of CMF ($n = 34$) at a standard dosage schedule. The major sites of metastatic disease included bone ($n = 24$), pulmonary ($n = 8$), bone and pulmonary ($n = 7$) and visceral ($n = 16$).

Patients unassessable for response by UICC (Hayward *et al.*, 1979) criteria ($n = 2$) or who died within 6 weeks ($n = 8$) were excluded from the analysis. Two patients remained biochemically unassessable throughout the study period. To qualify for an objective response (complete/partial) or static disease the minimum duration was considered 6 months (British Breast Group, 1974). Comparisons were made between assessments by UICC criteria with a minimum duration of remission or statis disease of 6 months and the changes in the biochemical markers measured at 6–8 and 12–16 weeks. In analysing the correlation between biochemical marker movement and UICC assessed response, objective responders and static disease were combined into a non-progressive disease group and compared with those patients that showed disease progression.

### *Biochemical assessment of response*

The biochemical score was calculated in the same manner as described for the first-line endocrine studies (Williams *et al.*, 1990; Robertson *et al.*, 1992). Namely, any change in marker whilst the patient is on therapy is related to the pretreatment value. A cut-off for each marker of the mean + 2 s.d. of the normal controls was calculated. Patients who never showed an elevation of the marker above this level were regarded as biochemically unassessable for that particular marker. Patients with an initial pre-treatment value below the cut-off which subsequently rose above the cut-off or patients with an initial value above the cut-off which subsequently increased above the inter-assay coefficient of variation (i.e., >10%) were regarded as showing an increasing marker level (scored +2), indicative of biochemical progression. Patients who started with an initially high value which fell to below the cut-off or patients with an initial value above the cut-off which subsequently decreased by more than the inter-assay coefficient of variation for that marker (i.e., >10%) were regarded as showing a decreasing marker level (scored −2), indicating a biochemical response; ESRs falling by >10% were scored −1. Patients with levels which started and remained above the cut-off but which moved by less than the inter-assay coefficient of variation were regarded as being biochemically stable and scored +1. These changes and the scoring attached to them are summarised in Table I. Scores for the individual markers were then added together to produce an overall biochemical index. Total scores >0 were considered as biochemical progressors whilst an index score of ⩽0 was considered a biochemical response.

### *Statistical analyses*

Actuarial survival – response duration analysis was performed using the statistical package SPSSX-21 (SPSS Inc.,

Correspondence: A.R. Dixon, Unilever Research Fellow, Professorial Unit of Surgery, City Hospital, Hucknall Road, Nottingham NG5 6JE, UK.
Received 26 June 1992; and in revised form 1 March 1993.

1986) life table analysis which calculates Gehan's generalised Wilcoxon rank test for censored data (Lee & Desu, 1972). Response/remission duration and survival were calculated from first treatment. Chi-squared analysis with Yates correction where appropriate was used to compared frequencies of integers between two variables. Paired independent measurements of continuous data were analysed using the Wilcoxon signed ranks sum test. Unpaired data were examined using the non-parametric Mann-Whitney U test.

*Serum markers*

ESR was measured within normal laboratory hours on the day of venepuncture using the Westergren technique. Serum markers were not measured immediately for practical purposes. All samples were assayed in duplicate and blind of clinical information on aliquots frozen and thawed once. Ca 15.3 was measured using the commerically available two site radio-immunoassay CIS ELSA kit® (CIS UK, High Wycombe). Despite the kit containing standards up to 250 U $ml^{-1}$, linearity was only demonstrable between 30 and 140 U $ml^{-1}$. The inter-assay coefficients of variation (CV) was 9.2%. CEA was measured using an in-house Unilever ELISA utilising a magnetically responsive solid phase, the monoclonal antibody 85A12 and a biotinylated detector polyclonal antibody – alkaline phosphatase-streptavidin conjugate. Intra- and inter-assay CVs were 6.2% and 8% respectively.

*Prospective clinical study using markers to direct therapy*

A small pilot prospective study was undertaken to examine the biochemical index in directing patient therapy. Twenty-six patients (19 from previous study group, seven additional patients) who had achieved objective UICC and a tumour marker assessed response following 6 months of first-line cytotoxic therapy were randomised to:

(1) Discontinued cytotoxic therapies ($n = 13$) and change to tamoxifen until clinical evidence of disease progression (control group).
(2) Continue the same cytotoxic regimen ($n = 13$) until the serum tumour markers rose by >10% of the trough value or moved out of the normal range (marker directed) at which point therapy would be changed.

All 26 patients were receiving 28 day cycles of a standard CMF regimen at the time of randomisation. Patients randomised to the tumour marker directed group were all made fully aware of the experimental nature of the proposed protocol and each gave written informed consent; they were allowed to discontinue at their own request or if the supervising clinician thought it was in their best interest. Dosage schedules were guided by serial blood counts and continued whilst the WBC count was $>2,500\,mm^{-3}$ and platelets $>100,000\,mm^{-3}$. After receiving 12 months of cytotoxics the patients were given the option of continuing with oral CMF: cyclophosphamide 150–200 mg (Mondays), methotrexate 25–50 mg (Wednesdays) and 5-fluoruracil 250–500 mg (Fridays), again dependent upon haematological indices. Five of the 13 patients in the control group received further cytotoxics upon later disease progression. Tumour marker estimations were performed at the time of study entry and repeated every 2 months. Three monthly UICC assessments were undertaken in the controls; X-rays were only requested in the marker directed group when clinically indicated. Disease progression and survival were calculated from the time of first treatment. Quality of life questionnaires in the form of a Rotterdam Symptom Checklist – RSCL (De Haes et al., 1990) were completed at study entry and every 3 months; the questionnaire assessed symptoms over the preceding 3 days. The RSCL contains three subscales: physical symptomatology due to disease and/or treatment (22 items); psychological symptoms (eight items) and activities of daily living (eight items). All items are rated on a four point scale (e.g. 'I feel tense', not at all (0); a little (1); somewhat (2); very much (3)). The psychological subscale yields a maximum score of 24.

The median age of the 13 patients under UICC/clinical direction (controls) was 58 years (range 41–69) in comparison to the 53 years (range 40–64) of the tumour marker directed (study) group. The principle sites of metastatic disease are shown in Table II.

### Results

UICC assessed response was compared with the biochemical index score comprised of CEA, CA 15.3 and ESR. The previously set cut-off values (see Table I) were used for all three markers (ie. CEA 6 ng $ml^{-1}$, CA 15.3 33 U $ml^{-1}$ and ESR 20 mm $h^{-1}$); marker changes from the baseline value of $>\pm 10\%$ were regarded as significant (Williams et al., 1990; Robertson et al., 1992). Using the three markers in combination 55 of 57 UICC assessable patients with systemic breast cancer were biochemically assessable (96%). A strong correlation ($P \leqslant 0.00$) was observed between the biochemical score calculated at 6–8 weeks and the UICC assessment at 3–4 months; sensitivity 89%; specificity 96%; positive predictive value 96%; negative predictive value 89% (Table III). A

**Table I** Scores for changes in marker concentrations

| | *Upper limit of normal* | *Normal limits* | *Decrease (>10%)* | *Stable (±<10%)* | *Increase (±>10%)* |
|---|---|---|---|---|---|
| CEA | 6 ng $ml^{-1}$ | 0 | −2 | +1 | +2 |
| CA 15.3 | 33 U $ml^{-1}$ | 0 | −2 | +1 | +2 |
| ESR | 20 mm $h^{-1}$ | 0 | −1 | +1 | +2 |

Individual markers scores are then added together to given the biochemical index score.

**Table II** Principal sites of metastatic disease in the prospective study of using markers to guide cytotoxic administration

| | *Control group (6/12 chemotherapy)* | *Study group (Continuous chemotherapy)* |
|---|---|---|
| Bone | 5 | 4 |
| Pulmonary | 2 | 1 |
| Bone and pulmonary | – | 3 |
| Visceral | 6 | 5 |

**Table III** CEA, ESR and CA 15.3 *vs* UICC response ($>\pm 10\%$)

(i) Pre-treatment *vs* 6–9 weeks in 55 assessable patients

| *3–4/12 UICC response* | *Biochemical Index Score* ≤0 | >0 |
|---|---|---|
| Response | 21 | 0 |
| Static | 5 | 3 |
| Progression | 1 | 25 |

$\chi^2 = 37.03$; 1 d.f.: $P = 0.0000$ (Combining response and static disease).
Sensitivity = 89%; Specificity = 96%; PPV = 96%; NPV = 89%

(ii) Pre-treatment *vs* 12–16 weeks in 55 assessable patients

| *3–4/12 UICC assessment* | *Biochemical Index Score* ≤0 | >0 |
|---|---|---|
| Response | 20 | 1 |
| Static | 4 | 4 |
| Progression | 3 | 23 |

$\chi^2 = 25.074$; 1 d.f.: $P = 0.0000$ (Combining response and static disease).
Sensitivity = 83%; Specificity = 88%; PPV = 89%; NPV = 82%

(iii) Pre-treatment *vs* 12–16 weeks in 55 assessable patients.

| *3/12 UICC assessment* | *Biochemical Index Score* ≤0 | >0 |
|---|---|---|
| Response | 21 | 0 |
| Static | 2 | 0 |
| Progression | 4 | 28 |

$\chi^2 = 37.569$; 1 d.f.: $P = 0.0000$ (Combining response and static disease).
Sensitivity = 100%; Specificity = 87%; PPV = 85%; NPV = 100%

PPV = +ve predictive value; NPV = −ve predictive value.

comparison of the 3 month biochemical score against the 3 and 6 months UICC assessment produced a similar significant correlation.

Of the 23 patients assessed as having non-progressive disease after 6 months of therapy, 100% had biochemical score ⩽0 at 12–16 weeks. Twenty-two patients (95%) had similar scores when the analysis was performed 6 weeks earlier. In contrast, 28 and 23 (84–87%) patients UICC assessed at 6 months as disease progressors had biochemical index scores >0 (biochemical progression) at 2 and 3 months respectively. Six of the eight patients assessed clinically (UICC) as having static disease following four cycles of cytotoxic therapy progressed during the following 3 months despite continuation of what was thought to be a clinically effective therapy; four of these patients were assessed biochemically at 2 months as disease progressors. These data suggests that an erroneous 3–4 month classification of stable disease can be avoided in some cases if the biochemical score is taken into account and so allow for an earlier change of cytotoxic regimens or simply palliation alone.

**Figure 1** Total quality of life scores for patients receiving either a 6/12 course or continuous chemotherapy whilst tumour markers continued to fall. (Mean and standard error bars).

*Prospective clinical study using markers to direct therapy*

Side effects were at a minimum in both treatment groups. Three patients in the control group had sufficient alopecia (WHO grade 3) during the initial 6 month course of cytotoxic to require a wig; a fourth developed a similar degree of alopecia in response to a course of doxorubicin upon subsequent relapse. Alopecia (WHO grade 3) was seen in two of the 13 patients randomised to the tumour marker directed group, this was despite maintenance cytotoxic therapy continuing for up to 26 months in some individuals. Four patients receiving maintenance cytotoxics experienced a rapid rejuvenation of initial hair loss (WHO grade 2–3) that had been sustained through a previous course of mitozantrone. Anticipatory vomiting complicated four cycles of maintenance cytotoxic therapy in one patient, nausea/vomiting (WHO grades 1–2) were reported by a further two. All 26 patients were prescribed the anti-emetic metaclopramide (10 mg 8 hourly) if required. Myelosuppression sufficient to require both a re-scheduling of planned cytotoxic administrations and dosage reduction occurred in three patients receiving maintenance chemotherapy, one of whom required a transfusion for anaemia; a single control patient required a blood transfusion prior to her fourth dose of mitozantrone. None of the 13 patients randomised to receive maintenance chemotherapy has requested to discontinue.

There were no differences in quality of life (QOL) total scores between the two groups upon entry into the study (Mann Whitney U statistic = 51; $P = 0.48$) or after the initial 6 month period of chemotherapy (Mann Whitney U statistic = 38.5; $P = 0.48$). The 6 month period of initial chemotherapy which had produced both an objective clinical and biochemical assessment of response in both sets of patients was also associated with an improved quality of life (falling threshold); the Wilcoxon matched-pair signed rank test statistic was 9 ($P = 0.030$) for controls and 0.0 ($P = 0.004$) for the study group. Whilst QOL total scores significantly increased (worsening quality of life) in the control group over the 3 month period following completion of cytotoxic therapy (Mann Whitney U statistic = 8.5; $P = 0.04$) before plateauing between 9 and 15 months, further improvements (falling scores) were recorded at 15 months in the group randomised to receive maintenance chemotherapy under tumour marker direction (Mann Whitney U statistic = 7.5; $P = 0.05$). All but two patients in the tumour marker directed (study) group recorded total scores within the normal range at 12 months; this compared to only three controls. Total quality of life scores with standard error bars are shown in Figure 1.

Despite the small number of patients entered into this preliminary study, a statistically significant difference ($P <$ 0.00) was observed in the period of clinical remission duration/disease control in favour of the marker guided/continuous chemotherapy group (median 21 months) compared to the patients randomised to discontinue cytotoxics (median 12 months) after 6 months (Figure 2). The median time to biochemical progression (17 months) for those who received marker directed treatment was significantly lengthened ($P =$ 0.05) over the time to clinical disease progression in the control group who discontinued chemotherapy. The observed advantages of a longer clinically apparent remission duration and improved quality of life were carried through into improved patient survival ($P = 0.04$) as calculated from the time of first treatment; 27 months compared to 17 months (Figure 3).

### Discussion

We have confirmed that the biochemical index derived for patients receiving first-line endocrine therapy (Williams *et al.*, 1990; Robertson *et al.*, 1992) is also applicable to patients treated by chemotherapy. A highly significant correlation was observed between the 3 and 6 month UICC response assessments and changes in biochemical index (CEA, CA 15.3 and ESR) calculated after 6–8 and 12–16 weeks of chemotherapy. The data also suggest that a 3 month classification of stable disease is not a clinically useful categorisation as most of these patients have an early relapse on continuation of the same cytotoxic. Early relapse may be avoidable if the assessment is made using the biochemical index and so allow for an earlier change of therapy.

Changes in the three markers appears to reflect the dynamics of a changing tumour mass in response to therapy in contrast to the UICC criteria which reflect structural changes. Potentially this would allow for the adoption of a more rational approach in deciding when to change or continue systemic therapies in that they provide an objective early measure of response or therapeutic failure. Marker estimations can also be performed quickly in a cost effective manner. One problem that has not been solved in measuring mucin epitopes is shedding. Epitopes of the mucins may be hidden and become available during chemotherapy. The phenomenon of spiking after the onset of chemotherapy is known in the case of CEA and probably also occurs with CA 15.3. In general this only takes some weeks. The three false negative biochemical scores >0 at 6–9 weeks were associated with 6 weeks estimations in patients receiving mitozantrone. Markers appear to have a potentially major role in the selection of 'true' objective responders to chemotherapy. If these people can be successfully identified the continued administration of cytotoxic therapy, whilst tumour marker levels continue to fall, appears to be advantageous.

Significant survival benefit is now well established for adjuvant chemotherapy. In contrast it has proved much harder to show that chemotherapy prolongs survival for advanced disease. This dilemma lies at the root of controver-

**Figure 2** Duration of clinical response for control and study patients and biochemical progression in the marker directed group.

**Figure 3** Survival of control and continuous chemotherapy (marker directed) groups from first treatment.

sies about its role in metastatic cancer. In 1980, Powles *et al.*, examined survival patterns in a group of patients, 50% of which had received combination chemotherapy post-1974 and compared them to a similar group treated pre-1974 (a period during which only 24% received combination chemotherapy). No significant difference was seen between the two groups, indeed the earlier group had, overall, a longer survival. The authors stated that their clinical observations suggested that some aptients with life-threatening visceral metastases did in fact have a survival benefit but that other patients, notably those with more indolent disease, may merely have suffered the toxicity and side effects of chemotherapy without any of the benefits. These observations have been confirmed by others (Paterson *et al.*, 1982; Patel *et al.*, 1986).

There are surprisingly few published data on the optimum duration of chemotherapy. In a small trial (Smalley *et al.*, 1976) 24 patients who responded to five-drug combination chemotherapy were randomised to stop after 24 weeks or to continue indefinitely; no survival advantage was seen for patients on maintenance treatment. A similar trial comparing 6 and 12 month maintenance chemotherapy was carried out in 31 patients treated with CMF or MMM chemotherapy (Smith, I.E., personal communication, 1990). Again no significant difference was found in progression-free survival or overall survival. This trial also demonstrated the difficulty in maintaining long-term chemotherapy; of 15 patients randomised to receive continuous chemotherapy, only three managed to complete 12 courses, with the rest stopping because of progressive disease or cumulative haematological toxicity. Identical findings were reported in an earlier study (Hortobagyi *et al.*, 1981). The 100% reported in our small study may relate to our adoption of a more gentle less stringent regimen i.e., our use of oral CMF and occasionally 35 day cycles for IV regimens and its administration to responsive patients only.

In contrast to these findings, a large (308 patients) randomised Australian study (Coates *et al.*, 1987) compared three courses of combination chemotherapy (cyclophosphamide, methotrexate, 5-flurouracil and prednisolone) resuming only when disease progressed with the same treatment given continuously until relapse; the hope was that the intervals of freedom from cytotoxic chemotherapy would contribute to an improved quality of life. The tumour response rate (33%) for intermittent therapy was significantly lower than the 44% observed with continuous chemotherapy. Short duration chemotherapy was also associated with a significantly shorter time to disease progression and a trend towards a shorter survival, with relative risk of mortality of 1.4 (95% confidence interval 1.10–1.79; $P = 0.0007$). In addition, this trial showed that short duration chemotherapy was associated with poorer overall quality of life score as assessed by symptom control, mood and general well-being. As expected, no differences were seen between the two patient groups during the first three cycles. It is important to note that the continuous policy yielded superior quality of life even in patients whose best tumour response was stable disease as well as those whose tumour responded. Further supportive evidence is provided by a randomised Canadian trial (Tannock *et al.*, 1988) comparing two dose levels of CMF. As anticipated, LASA scores showed a trend towards more nausea and significantly greater hair loss for patients receiving high dose treatment. No differences were detected for vomiting, mucositis or diarrhoea. However, those patients receiving the higher dose schedule showed a trend towards better symptom relief and general well-being including pain, mobility housework, anxiety and improved social life. This improvement correlated with patients on the higher dosage schedule achieving better response rates (30% *vs* 11%) and significantly improved survival (16 months *vs* 13 months).

Overall it would seem that the beneficial effects of cytotoxic chemotherapy in controlling disease outweigh the negative effect of treatment related side-effects and result in an improvement in quality of life as perceived by the patient. These findings are in accord to those reported within this paper. The described trial does not compare tumour marker directed chemotherapy against clinical directed chemotherapy but rather continuous *vs* intermittent chemotherapy. The value of the tumour markers was in selecting the study population i.e., only patients who had shown an objective biochemical response following 6 months administration of cytotoxics. If we can truly identify chemosensitive – responding patients and continue prescribing maintenance chemotherapy whilst the response continues then further improvements in quality of life, disease stabilisation and survival may be anticipated; tumour markers offer the attending clinician this ability. Fears of excess financial costs from biochemical monitoring were not substantiated in a subsequent study (unpublished findings).

If these findings are substantiated, the nihilistic approach frequently adopted by many clinicians to the treatment of this disease wil need reconsideration. A logical continuation of this study is to examine continuous chemotherapy (marker directed) in biochemical responders and compare against continuous chemotherapy whilst tolerable in UICC responders (clinical directions); such a trial is currently underway.

A.R. Dixon is funded by Unilever Research, Colworth Laboratory, Sharnbrook, Bedford.

### References

BRITISH BREAST GROUP (1974). Assessment of response to treatment in advanced breast cancer. *Lancet*, **ii**, 38–39.

COATES, A., GEBSKI, V., BISHOP, J.F., JEAL, P.N., WOODS, R.I., SYNDER, R., TATTERSALL, M.H.N., BYRNE, M., HARVEY, V., GILL, G., SIMPSON, J., DRUMMOND, R., BROWNE, J., VAN COOTON, R. & FORBES, J.F. (1987). Improving the quality of life during chemotherapy for advanced breast cancer: a comparison of intermittent and continuous treatment strategies. *N. Engl. J. Med.*, **317**, 1490–1495.

COOMBES, R.C., GAZET, J.C., SLOANE, S.C.H., POWLES, T.J., FORD, H.T., LAWRENCE, D.J.R. & NEVILLE, A.M. (1977). Biochemical markers in breast cancer. *Lancet*, **ii** 132–134.

COVE, D.H., WOODS, K.L., SMITH, S.C.H., BURNETT, D., LEONARD, J., GRIEVE, R.J. & HOWELL, A. (1979). Tumour markers in breast cancer. *Br. J. Cancer*, **40**, 710–718.

COWEN, D.M., SEARLE, F., WARD, A.M., BENSON, E.A., SMIDDY, F.G., EAVES, G. & COOPER, E.H. (1978). Multivariate biochemical indicators of breast cancer: an evaluation of their potential in routine practice. *Eur. J. Cancer*, **14**, 885–893.

DE HAES, J.C.J.M., VAN KNIPPENBERG, F.C.E. & NEIJT, J.P. (1990). Measuring psychological and physical distress in cancer patients: structure and application of the Rotterdam Symptom Checklist. *Br. J. Cancer*, **62**, 1034–1038.

FRANCHIMONT, P., ZANGERLE, P.F., NOGAREDE, J., BURRY, J., MOLTER, F. & REVETER, A. (1976). Simultaneous assays of cancer associated antigens in various neoplastic disorders. *Cancer*, **38**, 2287–2295.

HAYWARD, J.L., CARBONE, P.P., HEUSON, J.C., KUMAOKA, S., SEGALOFF, A. & RUBENS, R.D. (1979). Assessment of response to therapy in advanced breast cancer: a project of the Programme on Clinical Oncology of the International Union against Cancer, Geneva, Switzerland. *Cancer*, **39**, 1289–1294.

HORTOBAGYI, G.N., BLUMENSCHEIN, G.R., BUZDAR, A.U., YAP, H.Y., SCHELL, F.C., BARNES, B.C. & BURGESS, M.A. (1981). Combination chemotherapy with FAC-BCG for metastatic breast cancer: the impact of CMF maintenance chemotherapy. *J. Surg. Oncol.*, **18**, 163–172.

LEE, E.T. & DESU, M.M. (1972). A computer programe for comparing k samples with right censored data. *Computer Programms in Biomed.*, **2**, 315–321.

PATEL, J.K., NEMOTO, T., VEZERDIS, M., PETRELLI, N., SUH, O. & DAO, T.L. (1986). Does more intensive palliative treatment improve survival in advanced breast cancer. *Cancer*, **57**, 567–570.

PATERSON, A.H.G., SZAFRAN, O., CORNISH, F., LEES, W. & HANSON, J. (1982). Effect of chemotherapy on survival in advanced breast cancer. *Breast Cancer Res. Treat.*, **1**, 357–363.

POWLES, T.J., SMITH, I.E., FORD, H.T., COOMBES, R.C., JONES, J.M. & GAZET, J.-C. (1980). Failure of chemotherapy to prolong survival in a group of patients with metastatic breast cancer. *Lancet*, **1**, 580–582.

ROBERTSON, J.F.R., PEARSON, D., PRICE, M.R., SELBY, C., BLAMEY, R.W. & HOWELL, A. (1992). Objective measurement of therapeutic response in breast cancer using tumour markers. *Br. J. Cancer*, **64**, 757–761.

SMALLEY, R.V., MURPHY, S., HUGHLEY, C.M. & BARTOLUCCI, A.A. (1976). Combination versus sequential five drug chemotherapy in mestatatic carcinoma of the breast. *Cancer Res.*, **36**, 3911–3916.

SMITH, I.E. (1990). Personal communication.

SPSS INC. (1986). *SPSS* User's Guide, McGraw-Hill: New York.

TANNOCK, I.F., BOYD, N.F., DEBOER, G., ERLICHMAN, C., FINE, S., LAROLQUE, G., HAYES, C., PERRAULT, D. & SUTHERLAND, H. (1988). Randomised trial of two dose levels of cyclophosphamide, methotrexate and flurouracil chemotherapy for patients with metastatic breast cancer. *J. Clin. Oncol.*, **6**, 1377.

WILLIAMS, M.R., TURKES, A., PEARSON, D., GRIFFITHS, K. & BLAMEY, R.W. (1990). An objective biochemical assessment of therapeutic response in metastatic breast cancer a study with external review of clinical data. *Br. J. Cancer*, **61**, 126–132.